# Romosozumab Improves Tissue Thickness–Adjusted Trabecular Bone Score in Women With Osteoporosis and Diabetes

**DOI:** 10.1210/clinem/dgae862

**Published:** 2025-01-24

**Authors:** Serge Ferrari, Donald Betah, Robert G Feldman, Bente L Langdahl, Mary Oates, Jen Timoshanko, Zhenxun Wang, Ruban Dhaliwal

**Affiliations:** Geneva University Hospital, Geneva 1211, Switzerland; Amgen Inc., Thousand Oaks, CA 91320, USA; MemorialCare Saddleback Medical Center, Laguna Hills, CA 92653, USA; Aarhus University Hospital and Aarhus University, 8200 Aarhus, Denmark; Amgen Inc., Thousand Oaks, CA 91320, USA; UCB, Slough SL1 3WE, UK; Amgen Inc., Thousand Oaks, CA 91320, USA; The University of Texas Southwestern Medical Center, Dallas, TX 75390, USA; Massachusetts General Hospital, Boston, MA 02114, USA

**Keywords:** anabolic, DXA, bone mineral density, fracture risk, type 2 diabetes (T2D), osteoporosis

## Abstract

**Context:**

Trabecular bone score (TBS), a gray-level texture index derived from lumbar spine (LS) dual-energy x-ray absorptiometry (DXA) scans, is decreased in patients with diabetes and is associated with increased fracture risk, independent of areal bone mineral density (aBMD), but potentially influenced by abdominal fat tissue.

**Objective:**

Evaluate effect of romosozumab (210 mg monthly) for 12 months followed by alendronate (70 mg weekly) for 24 months vs alendronate alone (70 mg weekly) for 36 months on LS aBMD and TBS in women with type 2 diabetes (T2D) enrolled in the ARCH study.

**Methods:**

This post hoc analysis included women from ARCH who had T2D at baseline and LS DXA scans at baseline and ≥1 postbaseline visit (romosozumab-to-alendronate, n = 165; alendronate-to-alendronate, n = 195). aBMD and TBS (determined by an updated tissue thickness–adjusted TBS algorithm [TBS_TT_]) were assessed on LS DXA scans at baseline and ≥1 postbaseline visit (months 12, 24, and 36).

**Results:**

Romosozumab led to significantly greater gains in LS aBMD and TBS_TT_ at month 12 vs alendronate, and the greater gains with romosozumab were maintained after transition to alendronate and persisted significantly at months 24 and 36 vs alendronate alone. TBS_TT_ percentage changes weakly correlated to LS aBMD percentage changes from baseline to month 36 (romosozumab-to-alendronate, *R^2^* = 0.1493; alendronate-to-alendronate, *R^2^* = 0.0429).

**Conclusion:**

In postmenopausal women with osteoporosis and T2D, 12 months of romosozumab followed by 24 months of alendronate vs alendronate alone significantly improved LS aBMD and TBS_TT_ (independently of abdominal fat) and to a greater extent. Hence, romosozumab may improve bone strength in patients with T2D.

**Trial registration:**

ClinicalTrials.gov—NCT01631214

Type 2 diabetes (T2D) is associated with low bone turnover, reduced bone strength, and increased fracture risk, despite relatively preserved areal bone mineral density (aBMD) (1-3). Several studies using high-resolution peripheral quantitative computed tomography have shown that in T2D, trabecular bone volume and microarchitecture are preserved, whereas cortical porosity may be increased, at least when microvascular complications are present (1, 4-8). On another side, trabecular bone score (TBS), which is derived from lumbar spine (LS) dual energy x-ray absorptiometry (DXA) scans and is an indirect measure of trabecular bone microarchitecture (9, 10), has been reported to be decreased in patients with diabetes mellitus and associated with increased fracture risk, independent of hip aBMD (11) as measured by DXA. TBS uses the gray-level texture of 2-dimensional DXA images to characterize the variations in gray-level amplitude in the corresponding 3-dimensional tissue microarchitecture (9, 10). Since TBS is negatively influenced by abdominal fat, this could at least partly explain why TBS appears lower in patients with T2D compared with those without diabetes or those with normal glucose levels (12); moreover, observed longitudinal changes in TBS have been predominantly explained by changes in tissue thickness and weight change (13).

A recently updated tissue thickness–adjusted TBS algorithm (TBS_TT_) has been developed that takes into account regional LS soft tissue thickness measured directly from the DXA system (14). The updated TBS_TT_ has been shown to mitigate regional soft tissue noise on DXA images better than TBS corrected for body mass index (TBS_BMI_) and overcome the residual negative correlation of TBS with body size and composition parameters (14). A recent cohort study showed that TBS_TT_ was higher in patients with T2D compared with those without (15).

Currently, there are no reports from interventional trials with an endpoint to evaluate fracture risk reduction in patients with T2D and bone fragility. However, post hoc analyses of randomized controlled trials comparing antiresorptives with placebo in mostly postmenopausal women with osteoporosis have generally shown similar reductions in vertebral and nonvertebral fractures in subsets of patients with diabetes compared with those without (16). Considering that bone fragility in diabetes not only stems from altered bone mineralization but also changes in bone microarchitecture, treatment with osteoanabolic drugs is particularly appealing. Romosozumab treatment in postmenopausal women with osteoporosis has been shown to increase aBMD and decrease the risk of fractures compared with placebo or alendronate (17-20). In the Active-Controlled Fracture Study in Postmenopausal Women With Osteoporosis at High Risk (ARCH) trial, monthly subcutaneous injections of romosozumab for 12 months followed by 24 months of open-label alendronate led to significantly improved bone mass and bone strength parameters and superior risk reduction in new vertebral, clinical, nonvertebral, and hip fractures vs alendronate alone (20, 21). Further, in a subset of patients from ARCH, romosozumab significantly improved TBS_TT_ and TBS_BMI_ vs alendronate alone (22).

Here, we evaluated the effect of 12 months of romosozumab followed by 24 months of alendronate vs 36 months of alendronate alone on LS aBMD and TBS_TT_ in a post hoc analysis of LS DXA scans collected from a subgroup of patients with T2D from the ARCH study.

## Materials and Methods

### Study Design and Patients

**Figure 1.**
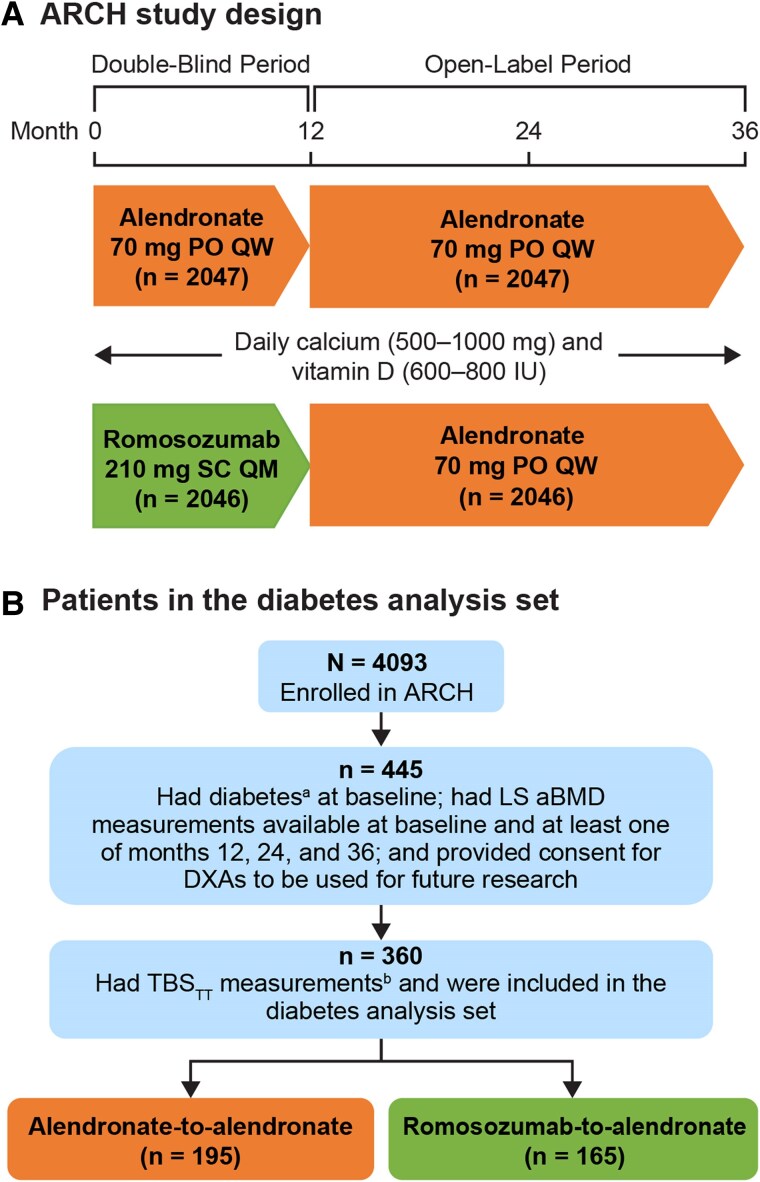
Study design and diabetes analysis set. Eligibility criteria for ARCH included age 55 to 90 years and 1 of the following: T-score ≤−2.5 at the total hip or femoral neck and either ≥1 moderate or severe vertebral fracture or ≥2 mild vertebral fractures or T-score ≤−2.0 at the total hip or femoral neck and either ≥2 moderate or severe vertebral fractures or a fracture of the proximal femur sustained 3 to 24 months before randomization. Of the 195 patients who received blinded alendronate, 99.5% (194/195) had T2D and 0.5% (1/195) had T1D; of the 165 patients who received blinded romosozumab, 98.8% (163/165) had T2D and 1.2% (2/165) had T1D. ^a^Had a medical history of hyperglycemia/new-onset diabetes mellitus with only narrow scope terms at baseline. ^b^Patients with BMI >38 kg/m^2^ or <15 kg/m^2^ were excluded as that was out of the range for a proper TBS assessment; patients were also excluded if the DXA system or scan acquisition mode used for aBMD assessment was not compatible with TBS computation. Abbreviations: aBMD, areal bone mineral density; BMD, bone mineral density; BMI, body mass index; DXA, dual-energy x-ray absorptiometry; LS, lumbar spine; PO, orally; QM, monthly; QW, weekly; SC, subcutaneously; T1D, type 1 diabetes; T2D, type 2 diabetes; TBS, trabecular bone score; TBS_TT_, tissue thickness–adjusted TBS.

ARCH (NCT01631214) was an international, multicenter, randomized, double-blind, active-controlled, phase 3 study in 4093 postmenopausal women with osteoporosis and a prior vertebral or hip fracture (Fig. 1A), and the study design was previously published (21). In the double-blind period that lasted for 12 months, patients were randomized 1:1 to receive monthly subcutaneous romosozumab 210 mg or weekly oral alendronate 70 mg. After completion of the double-blind period, all patients received open-label weekly oral alendronate 70 mg for a period of ≥12 months until the end of the event-driven study (clinical fractures had been confirmed in ≥330 patients and all patients had completed the month 24 visit), with a median follow-up of 2.7 years from patient enrollment (21). Patients received daily supplementation of calcium and vitamin D.

ARCH was conducted in compliance with the Declaration of Helsinki, International Council for Harmonization Guidelines for Good Clinical Practice, and local country regulations. Study protocols were approved by each center's institutional review board or independent ethics committee. Patients provided written informed consent before study initiation.

Patients were selected for this post hoc TBS_TT_ analysis based on a 2-step process (Fig. 1B). Of the 4093 patients enrolled in the ARCH study, a subset of 445 patients (212 in the romosozumab group and 233 in the alendronate group) had diabetes (defined as a medical history of hyperglycemia/new-onset diabetes mellitus with only narrow scope terms) at baseline (as self-reported by patients) and LS aBMD measurements at baseline and ≥1 postbaseline visit (months 12, 24, and 36) and had provided consent for DXA scans to be used for future research. Of the 445 patients, 4 (0.9%) had type 1 diabetes (T1D) and 441 (99.1%) had T2D. TBS evaluation excluded 85 patients because they had a body mass index (BMI) of more than 38 kg/m^2^ or less than 15 kg/m^2^, which strictly fall outside of the range recommended by the manufacturer, or because the DXA system (eg, the outdated models of Lunar DPX, DPX-NT, or Hologic Explorer scanners with inferior image resolutions and signal-to-noise ratios of >1 mm) or scan acquisition mode (eg, “express” mode on a Hologic scanner) used for aBMD assessment was not compatible with TBS computation. Based on these criteria, 360 patients were found to have valid TBS measurements and were included in the TBS_TT_ analysis of patients with diabetes in the ARCH study (3 [0.8%] with T1D and 357 [99.2%] with T2D).

### Study Assessments

For aBMD measurements, LS DXA scans were performed on 4 vertebrae (L1–L4) using Lunar (GE Medical Systems, Madison, WI, USA) or Hologic (Hologic, Inc., Bedford, MA, USA) DXA bone densitometers at baseline and months 12, 24, and 36. Fractured or clearly abnormal and nonassessable vertebrae were excluded from both aBMD and TBS analyses in accordance with the recommendations from the International Society for Clinical Densitometry (23), with all analyses including ≥2 evaluable vertebrae. DXA scans were centrally analyzed by Clario (formerly BioClinica or Synarc; Newark, CA, USA) while blinded to treatment assignments (18, 20, 21). TBS_TT_ was assessed on the previously obtained LS DXA scans in the same region of interest as aBMD, using the TBS_TT_ computation algorithm (TBS software 4-beta [Medimaps Group, Geneva, Switzerland]) (14). As for aBMD, all TBS analyses were performed blinded from clinical outcomes and treatment assignments.

### Statistical Analyses

Patient data included LS aBMD and TBS_TT_ measurements at baseline and ≥1 postbaseline visit. LS aBMD and TBS_TT_ data were analyzed based on a repeated measures model adjusting for treatment, presence of severe vertebral fracture at baseline, visit, treatment-by-visit interaction, and baseline aBMD or TBS value as fixed effects, with DXA machine type and baseline aBMD or TBS value-by-machine type interaction as covariates. Statistical inferences on differences between the romosozumab-to-alendronate and alendronate alone groups were assessed at each time point. Results were reported as least squares means with their respective 2-sided 95% confidence intervals.

Bone outcomes according to predefined TBS_TT_ categories derived only for the current study population (TBS_TT_ >1.074, normal; TBS_TT_ >1.027 to ≤1.074, partially degraded; and TBS_TT_ ≤1.027, degraded) were evaluated at baseline and months 12, 24, and 36 in each treatment group. The TBS_TT_ thresholds were derived from the tertile analysis of the same study population from an individual-level meta-analysis (24). Bhapkar's test was used to check whether these marginal proportions were homogeneous.

The correlations between percentage change from baseline in LS aBMD and TBS_TT_ were evaluated using Pearson correlation coefficients at months 12, 24, and 36 within each treatment group.

All statistical tests were 2-tailed, with *P* ≤ .05 set as the threshold for statistical significance. All *P*-values were nominal and not adjusted for multiplicity.

## Results

### Patients and Baseline Characteristics

The post hoc subpopulation of the ARCH study comprised 360 postmenopausal women with osteoporosis and diabetes; 99.2% (357/360) had T2D and 0.8% (3/360) had T1D. Of these, 165 patients (98.8% [163/165] with T2D; 1.2% [2/165] with T1D) received blinded romosozumab for 12 months, followed by open-label alendronate (romosozumab-to-alendronate group) and 195 patients (99.5% [194/195] with T2D; 0.5% [1/195] with T1D) received alendronate (alendronate-to-alendronate group). Baseline characteristics were similar between the romosozumab-to-alendronate and alendronate-to-alendronate alone groups (Table 1). Baseline characteristics of the 2 groups in the T2D analysis subpopulation were also similar to those of the overall ARCH study population (Table 1) (21). In the T2D analysis subpopulation, mean age was 75.5 years, and most women had a prior osteoporotic fracture at or after the age of 45 years (98.6%) and a prevalent vertebral fracture at baseline (93.6%); the percentage of patients with previous nonvertebral fracture at baseline was 37.2% (41.8% in the romosozumab-to-alendronate group and 33.3% in the alendronate-to-alendronate group). Mean LS aBMD T-scores and TBS_TT_ between the romosozumab and alendronate groups did not differ substantially.

**Table 1.** Baseline characteristics

Characteristic	ARCH diabetes substudy population		ARCH overall population	
	Alendronate n = 195*^a^*	Romosozumab n = 165*^a^*	Alendronate n = 2047	Romosozumab n = 2046
Age, years, mean ± SD	75.2 ± 7.1	75.7 ± 7.3	74.2 ± 7.5	74.4 ± 7.5
Body mass index, kg/m^2^, mean ± SD	25.9 ± 4.5	26.6 ± 4.3	25.4 ± 4.4	25.5 ± 4.4
Race, n (%)				
White or Caucasian	116 (59.5)	108 (65.5)	1415 (69.1)	1447 (70.7)
Asian, American Indian/Alaska Native, Hawaiian Native/Other Pacific Islander	27 (13.8)	13 (7.9)	158 (7.7)	142 (6.9)
Black or African American	4 (2.1)	4 (2.4)	23 (1.1)	19 (0.9)
Multiple, other	48 (24.6)	40 (24.2)	450 (22.0)	438 (21.4)
aBMD T-score, mean ± SD				
Lumbar spine	−2.9 ± 1.3	−2.6 ± 1.3	−3.0 ± 1.2	−2.9 ± 1.3
Total hip	−2.8 ± 0.7	−2.6 ± 0.7	−2.8 ± 0.7	−2.8 ± 0.7
Femoral neck	−2.9 ± 0.5	−2.8 ± 0.4	−2.9 ± 0.5	−2.9 ± 0.5
Previous osteoporotic fracture at ≥45 years of age, n (%)	193 (99.0)	162 (98.2)	2029 (99.1)	2022 (98.8)
Prevalent vertebral fracture, n (%)	184 (94.4)	153 (92.7)	1964 (95.9)	1969 (96.2)
Severe vertebral fracture	116 (59.5)	101 (61.2)	1321 (64.5)	1369 (66.9)
Previous nonvertebral fracture at ≥45 years of age, n (%)	65 (33.3)	69 (41.8)	770 (37.6)	767 (37.5)
TBS_TT_ (unitless), mean ± SD	1.010 ± 0.101	1.006 ± 0.104	1.004 ± 0.117*^b^*	1.015 ± 0.108*^b^*

n = number of patients who had TBS_TT_ measurements at baseline and ≥1 postbaseline visit for each treatment group in the ARCH diabetes substudy population or number of patients in each treatment group for the ARCH overall population.

Abbreviations: aBMD, areal bone mineral density; T1D, type 1 diabetes; T2D, type 2 diabetes; TBS, trabecular bone score; TBS_TT_, tissue thickness–adjusted trabecular bone score.

^*a*^Overall, 195 patients (99.5% [194/195] with T2D; 0.5% [1/195] with T1D) received alendronate alone for 36 months and 165 patients (98.8% [163/165] with T2D; 1.2% [2/165] with T1D) received blinded romosozumab for 12 months and then alendronate for 24 months.

^*b*^Mean values derived from a subset of 378 patients (alendronate: 188; romosozumab: 190) who had TBS_TT_ measurements in the ARCH overall population analyzed in the TBS study (McClung MR, et al *J Bone Miner Res*. 2024;zjae194).

### Between-Group Percentage Change From Baseline to Months 12, 24, and 36 in LS aBMD and TBS_TT_

**Figure 2.**
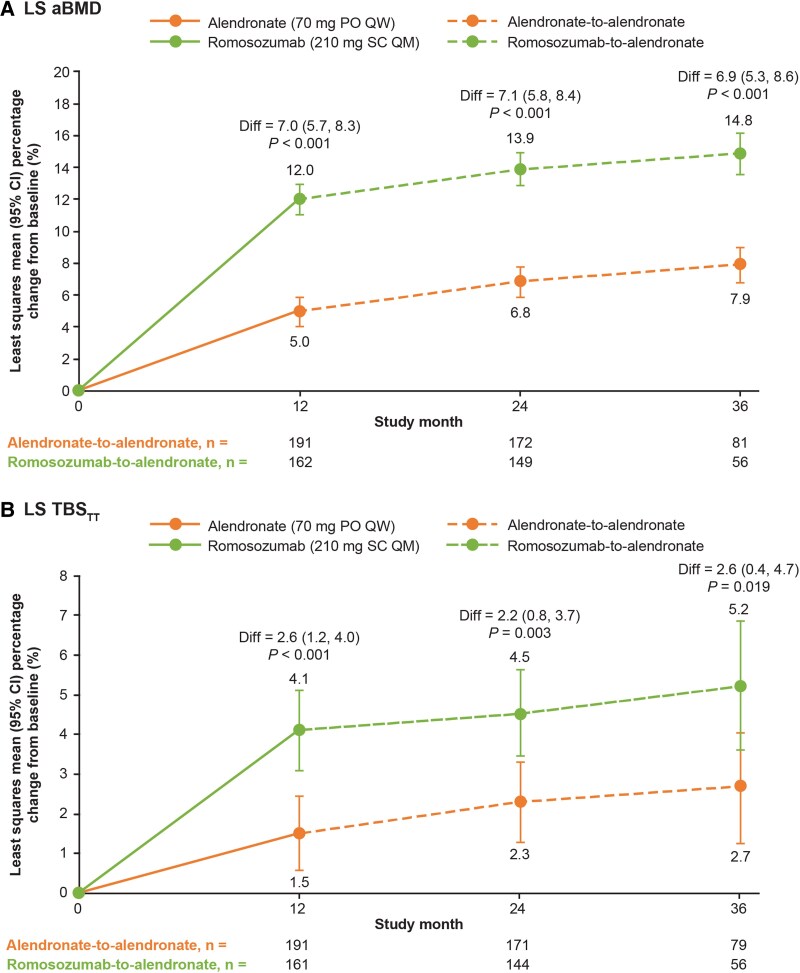
Percentage change from baseline to months 12, 24, and 36 by visit and treatment group for (A) LS aBMD and (B) LS TBS_TT_ in the T2D subgroup. n = number of patients with aBMD and TBS_TT_ measurements. The LS aBMD and TBS_TT_ data were analyzed based on a repeated measures model adjusting for treatment, presence of severe vertebral fracture at baseline, visit, treatment-by-visit interaction, baseline aBMD, or TBS value as fixed effects, with DXA machine type and baseline aBMD or TBS value-by-machine type interaction as covariates. Abbreviations: aBMD, areal bone mineral density; CI, confidence interval; Diff, percentage change from baseline for romosozumab treatment group minus percentage change from baseline for alendronate treatment group; DXA, dual-energy x-ray absorptiometry; LS, lumbar spine; PO, orally; QM, monthly; QW, weekly; SC, subcutaneously; T2D, type 2 diabetes; TBS, trabecular bone score; TBS_TT_, tissue thickness–adjusted trabecular bone score.

Significantly greater gains in LS aBMD were observed with romosozumab compared with alendronate over the 12 months of the double-blind treatment period, with a least squares mean difference of 7.0% (*P* < .001) at month 12 (Fig. 2A). The greater gains in LS aBMD with romosozumab persisted over the 24 months of the open-label alendronate period. The least squares mean differences between the romosozumab-to-alendronate and alendronate-to-alendronate groups were 7.1% (*P* < .001) at month 24 and 6.9% (*P* < .001) at month 36 (Fig. 2A).

Similarly, significant gains in LS TBS_TT_ were observed with romosozumab compared with alendronate over the 12 months of the double-blind treatment period—however, to a lesser extent, with a least squares mean difference of 2.6% (*P* < .001) at month 12 (Fig. 2B). The significant gains in LS TBS_TT_ with romosozumab persisted over the 24 months of the open-label alendronate period. The least squares mean differences between the romosozumab-to-alendronate and alendronate-to-alendronate groups were 2.2% (*P* = .003) at month 24 and 2.6% (*P* = .019) at month 36 (Fig. 2B).

### Within-Group Percentage of Patients by TBS_TT_ Risk Category at Baseline and Months 12, 24, and 36

**Figure 3.**
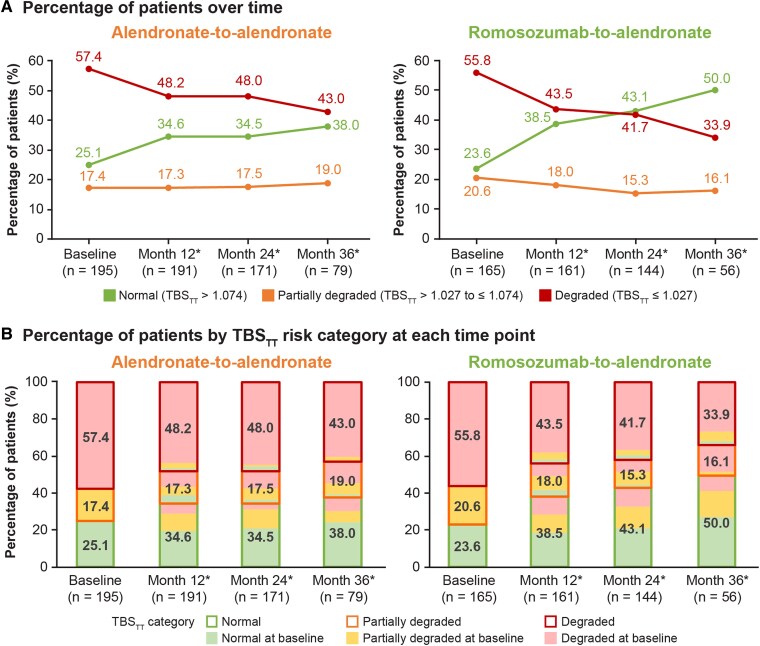
Percentage of patients by LS TBS_TT_ risk category at baseline and months 12, 24, and 36 in the alendronate-to-alendronate and romosozumab-to-alendronate groups in the subpopulation with T2D in ARCH (A) over time and (B) by TBS_TT_ risk category at each time point. n = number of women with mean calibrated TBS_TT_ measurements at baseline and months 12, 24, and 36. *P < .001 vs baseline based on Bhapkar's test for homogeneity. Abbreviations: LS, lumbar spine; T2D, type 2 diabetes; TBS_TT_, tissue thickness–adjusted trabecular bone score.

In the romosozumab-to-alendronate group, the percentage of women with “normal” TBS_TT_ values (TBS_TT_ >1.074) increased from 23.6% at baseline to 50.0% at month 36, that of women with “partially degraded” TBS_TT_ values (TBS_TT_ >1.207 to ≤1.074) decreased from 20.6% to 16.1%, and that of women with “degraded” TBS_TT_ values (TBS_TT_ ≤1.027) decreased from 55.8% to 33.9% (*P* < .001 for all categories and all timepoints) (Fig. 3A and 3B). A similar trend, albeit with smaller proportional changes, was observed in the alendronate-to-alendronate group; the percentage of women with “normal” TBS_TT_ values increased from 25.1% at baseline to 38.0% at month 36, that of women with “partially degraded” TBS_TT_ values slightly increased from 17.4% to 19.0%, and those with “degraded” TBS_TT_ values decreased from 57.4% to 43.0% (*P* < .001) (Fig. 3A and 3B).

### Relationship Between LS aBMD and TBS_TT_ Percentage Change From Baseline to Months 12, 24, and 36

**Figure 4.**
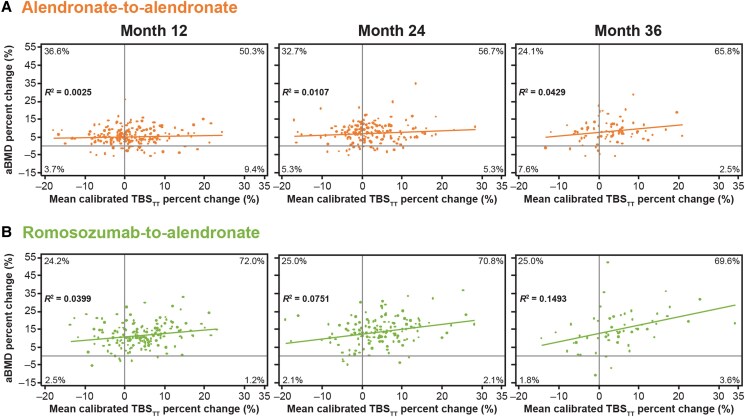
Relationship between LS aBMD and TBS_TT_ percentage change from baseline to months 12, 24, and 36 in (A) the alendronate alone group and (B) the romosozumab-to-alendronate group. Data analyzed for patients with aBMD and TBS_TT_ measurements at baseline and months 12, 24, or 36. Pearson correlation: *R*^2^ = 0.0025 at month 12, *R*^2^ = 0.0107 at month 24, and *R*^2^ = 0.0429 at month 36 in the alendronate alone group; *R*^2^ = 0.0399 at month 12, *R*^2^ = 0.0751 at month 24, and *R*^2^ = 0.1493 at month 36 in the romosozumab-to-alendronate group. Percentage of values are shown in each quadrant. Abbreviations: aBMD, areal bone mineral density; LS, lumbar spine; *R*^2^, correlation coefficient; TBS_TT_, tissue thickness–adjusted trabecular bone score.

Percentage changes from baseline in TBS_TT_ poorly correlated with those in LS aBMD, with correlations remaining low in both groups across the treatment period (*R^2^* = 0.0399 at month 12, *R^2^* = 0.0751 at month 24, and *R^2^* = 0.1493 at month 36 in the romosozumab-to-alendronate group; *R^2^* = 0.0025 at month 12, *R^2^* = 0.0107 at month 24, and *R^2^* = 0.0429 at month 36 in the alendronate-to-alendronate group) (Fig. 4).

## Discussion

In this post hoc analysis of postmenopausal women in the ARCH study who had osteoporosis, prior fracture, and T2D, 12 months of romosozumab followed by 24 months of alendronate significantly improved LS aBMD, and to a lesser extent LS TBS_TT_, compared with 36 months of alendronate alone. The major difference in both aBMD and LS TBS_TT_ between the 2 treatment groups occurred during the first 12 months when the first group received osteoanabolic therapy with romosozumab vs antiresorptive therapy with alendronate in the second group. These gains in aBMD and TBS_TT_ were maintained over the 24 months of open-label alendronate treatment in both groups. The increments in LS TBS_TT_ were small and largely independent of the increments in LS aBMD. While significantly more individual patients in both treatment groups achieved normal LS TBS_TT_ and fewer patients had degraded LS TBS_TT_ throughout treatment compared with baseline in both groups, the trend was greater in patients who received romosozumab first for 12 months followed by alendronate for 24 months than in women who received alendronate for the whole 36 months.

Baseline TBS_TT_ did not differ between the subgroup of patients with T2D analyzed in our current study and the subgroup of patients with TBS_TT_ measurements (which had only a few patients with diabetes) in the previously published study by McClung et al (22). Although both subgroups had small sample sizes, this observation may indicate that, once corrected for abdominal fat, TBS is actually not diminished in patients with diabetes. This question has now been reexamined in the large Manitoba cohort, showing indeed that TBS adjusted for abdominal thickness is not lower but higher in patients with diabetes (15).

Our results indicate that the subgroup of patients with T2D in the ARCH study responded to romosozumab with larger increases in LS BMD and TBS_TT_ with alendronate, with the TBS_TT_ results similar to those observed for a subpopulation of patients in ARCH mainly without diabetes (22). As in the overall population of the ARCH study (21), patients with diabetes treated with romosozumab responded with larger increases in LS BMD compared with patients treated with alendronate. These results are consistent with the findings from the imaging substudy of ARCH (20), which showed that for a given absolute change in integral volumetric BMD, a larger increase in bone strength as assessed by the finite element analysis of quantitative computed tomography scans was observed for the romosozumab-to-alendronate group compared with the alendronate-to-alendronate group, indicating that the change in vertebral bone strength may result not only from the gains in aBMD but also from improvements in BMD distribution or bone microarchitecture.

The greater and more rapid increases in aBMD and TBS_TT_ that we observed with romosozumab followed by alendronate compared with alendronate alone may contribute to a greater reduction in fracture risk in patients at high risk for fracture, such as postmenopausal patients with diabetes (21, 25). Previous studies have shown that TBS might also be differentially affected by various therapeutic agents, which is plausible given the different mechanisms of action of therapeutic agents for osteoporosis (9, 26-28). For example, gains in TBS or TBS_TT_ have been observed with bisphosphonates; however, these gains were smaller compared with gains observed with more potent antiresorptive agents, such as denosumab, and with bone-building agents, such as romosozumab and teriparatide (26-31).

Our analysis confirmed that changes in TBS_TT_ were largely independent of the percentage change in LS aBMD over 12, 24, or 36 months of treatment, with either romosozumab-to-alendronate or alendronate-to-alendronate. The correlation coefficients in both treatment groups were similar to what has been reported with TBS_TT_ for romosozumab (22), alendronate (22), and other osteoporosis therapies (29) in generally nondiabetes populations.

We are not aware of published TBS_TT_ thresholds, so to evaluate the effect of romosozumab on achieving normal LS TBS_TT_, we derived the TBS_TT_ thresholds from the tertile analysis of the same study population from an individual-level meta-analysis as described for the derivation of TBS thresholds by McCloskey et al (24). Similarly, individual-level meta-analysis data from large populations could be used to establish TBS_TT_ thresholds for use across studies.

The strength of our analysis is that it used data from a randomized controlled trial with standardized assessments for aBMD and bone parameters and that patient baseline characteristics and LS aBMD changes over 36 months of treatment in both treatment groups were similar to those reported in the overall ARCH study population. Additionally, the use of the updated TBS_TT_ algorithm reduced the artifact associated with the presence of regional soft tissue compared with the previous TBS correlated with body size and composition (14). Data included in this post hoc analysis were mostly from patients with T2D, which is the more prevalent type of diabetes and the disease type in which aBMD fails to capture fracture risk. Moreover, central obesity is a common finding in T2D; thus, our study findings of TBS_TT_ are highly relevant to this population. A number of study limitations must be taken into consideration when interpretating results from our analysis. This TBS post hoc analysis was conducted in a small subset (8.8%) of the overall population of the ARCH, raising the potential for imbalances in covariates between treatment groups, including treatments for hyperglycemia, which may influence skeletal outcomes. Although 99.2% of patients in the analyzed subset had T2D, a small proportion (0.8%) had T1D. Additionally, longitudinal data are limited to a median follow-up of 2.7 years, with less than half of the patients having LS aBMD/TBS_TT_ results at month 36. Further, the small sample sizes do not allow for the analysis of benefits on fracture reduction. Further, as enrollment of patients with or without diabetes was not a prespecified consideration for the original ARCH study, no proactive patient assessments or diagnostic tests to detect diabetes were performed at baseline or during the course of the study, and no information on the use of antidiabetic drugs was proactively collected. Finally, this post hoc analysis did not evaluate fracture risk or bone pain as study outcomes.

In conclusion, in postmenopausal women with osteoporosis and T2D, 12 months of romosozumab followed by 24 months of alendronate significantly improved LS aBMD and TBS_TT_ (independent of abdominal fat) and to a greater extent compared with 36 months of alendronate alone. These changes may reflect a greater improvement in bone strength observed with romosozumab vs alendronate in patients with T2D.

## Data Availability

Qualified researchers may request data from Amgen clinical studies. Complete details are available at the following: https://wwwext.amgen.com/science/clinical-trials/clinical-data-transparency-practices/clinical-trial-data-sharing-request/
